# Reliability of recovery heart rate variability measurements as part of the Lamberts Submaximal Cycle Test and the relationship with training status in trained to elite cyclists

**DOI:** 10.1007/s00421-023-05385-z

**Published:** 2024-01-10

**Authors:** Robert P. Lamberts, Teun van Erp, Alejandro Javaloyes, Maaike M. Eken, Nelleke G. Langerak, Nicholas Tam

**Affiliations:** 1 Division of Movement Science and Exercise Therapy (MSET), Department of Exercise, Sport and Lifestyle Medicine, Faculty of Medicine and Health Sciences, Stellenbosch University, Suidwal Road, Stellenbosch, 7601 South Africa; 2 Department of Sport Science, Sports Research Centre, Miguel Hernández University of Elche, Alicante, Spain; 3 Institute of Sport and Exercise Medicine (ISEM), Department of Exercise, Sport and Lifestyle Medicine, Faculty of Medicine and Health Sciences, Stellenbosch University, Tygerberg, South Africa, Stellenbosch University, Tygerberg, South Africa; 4 Department of Research, Sint Maartenskliniek, Nijmegen, The Netherlands; 5 Neuroscience Institute and Division of Neurosurgery, Faculty of Health Sciences, University of Cape Town, Cape Town, South Africa; 6 On AG, Sport Science Laboratory, Zurich, Switzerland

**Keywords:** HRV, HRR, Peak power, Endurance cycling performance, Training status, LSCT

## Abstract

**Purpose:**

To determine if post-exercise heart rate variability, in the form of logged transformed root mean square of successive differences of the R–R intervals (LnRMSSD) can be measured reliably during the recovery from a submaximal cycle test and what the relationship of LnRMSSD is with training status of the cyclists.

**Methods:**

Fourteen male cyclists participated in the reliability part for the study, which included performing six Lamberts Submaximal Cycle Test (LSCT), during which recovery LnRMSSD was measured over 30 s (LnRMSSD_30 s_), 60 s LnRMSSD_60 s_)and 90 s LnRMSSD_90 s_). In addition, fifty male and twenty female cyclists completed a peak power output (PPO) test (including *V*O_2peak_) and 40 km time trial (40 km TT) before which they performed the LSCT as a standardized warm-up. Relationships between the LnRMSSD and PPO, *V*O_2peak_ and 40 km TT time were studied.

**Results:**

Due to the design of the LSCT, submaximal heart and breathing rate were similar at the end of stage 3 of the LSCT, as well as during the recovery periods. The highest reliability was found in LnRMSSD_60 s_ (ICC: 0.97) with a typical error of the measurement (TEM: 5.8%). In line with this the strongest correlations were found between LnRMSSD_60 s_ and PPO (*r* = 0.93[male]; 0.85[female]), *V*O_2peak_ (*r* = 0.71[male]; 0.63[female];) and 40 km TT (*r* = – 0.83[male]; – 0.63[female]).

**Conclusions:**

LnRMSSD_60 s_ can be measured reliably after the LSCT and can predict PPO, *V*O_2peak_ and 40 km TT performance well in trained-to-elite cyclists. These findings suggest that recovery LnRMSSD can potentially play an important role in monitoring and fine-tuning training prescriptions in trained-to-elite cyclists.

## Introduction

Being able to monitor changes in training status is valuable, as it can assist coaches and athletes to optimize training prescription and prevent maladaptation to training. Two commonly used parameters to asses overall well-being are heart rate recovery (HRR) and heart rate variability (HRV), which are regulated through the interplay of the sympathetic and parasympathetic nervous system. As both HRR and HRV are able to reflect changes in training status (Lamberts et al. 2010; Buchheit et al. 2008; Vesterinen et al. 2017; Nuuttila et al. 2022b), they both gained popularity as monitoring parameters for coaches and athletes. Subsequently, several researchers have shown that HRR and HRV can also be used to optimize training prescription in athletes (Capostagno et al. 2014, 2021; Javaloyes et al. 2018, 2020; Nuuttila et al. 2022a), while HRR in combination with other physiological parameters is able to reflect a state of functional over-reaching (Roete et al. 2021).

In contrast to HRR, which is quite straightforward to measure and analyze (Daanen et al. 2012), HRV can be analyzed in either the time, frequency or non-linear domain (Malik et al. 1996). However, log-transformed root mean square of successive differences of the R–R intervals (LnRMSSD), which is part of the time domain, has become the most popular HRV index to monitor and fine-tune training prescription in athletes (Nuuttila et al. 2022a, b; Javaloyes et al. 2018, 2020; Vesterinen et al. 2016, 2017).

While HRV traditionally had to be measured under well-controlled conditions (e.g., in a rested state, seated or supine position, controlled breathing and for minimally 5 min) (Malik et al. 1996), more recently, several researchers have started using ultra-short LnRMSSD measurements (as short as 30 s) after submaximal exercise, to monitor training status in athletes (Munoz et al. 2015; Krejci et al. 2018; Forner-Llacer et al. 2020). Although the findings of these studies are promising and the reduced time frame will assist with the practical applicability, LnRMSSD in these studies was still measured in a rested state to assure stability in potential confounding factors, such as heart and breathing rate during the measurement.

However, as exercise intensity increases, variability in physiological parameters, such as heart rate, decreases (Lamberts et al. 2004, 2011a). This raises the question of whether LnRMSSD can be measured after submaximal exercise, as generally done for the measurement of HRR.

To our knowledge, no study to date has determined if LnRMSSD during the recovery from a standardized submaximal test can be measured reliably, how this relates to HRR measurements and if and how these LNRMSSD measurements relate to the training status of an athlete. Therefore, the first aim of this study was to determine the reliability of LnRMSSD after a standardized cycle test in trained-to-elite-level cyclists, while the second aim was to determine the relationship between this LnRMSSD measurement and the training status of this cyclist.

## Methods

### Experimental approach to the problem

The study protocol consisted of two parts, namely, *Part I*, which aimed to determine the reliability of the recovery LnRMSSD and Part II, which aimed to determine the relationships between the LnRMSSD measurements and the training status markers of trained-to-elite cyclists. The inclusion criteria for the study were that cyclists needed to have at least years of cycling experience and were training at least 6 h per week in 6 weeks before the study started (Lamberts 2014; Lamberts et al. 2011b). Before enrolling, all cyclists had to complete the physical activity readiness questionnaire (PAR-Q) and sign a written informed consent form.

As part of the study, all cyclists were familiarised with the Lamberts Submaximal Cycle Test (LSCT), Peak Power output (PPO) test and 40 km time trial (40 km TT) (Lamberts et al. 2009, 2011b). All participants were asked to refrain from eating and drinking any caffeine 2 h before the tests, while all tests were scheduled and conducted at the same time of the day (within 1 h). In addition, all tests were conducted under very strict and well-controlled environmental conditions (21.5 ± 0.6 °C, 51 ± 3% relative humidity, 102.1 ± 0.7 kPa). Height, body fat percentage (Durnin and Womersley 1974) and the sum of seven skinfolds (triceps, biceps, supra-iliac, sub-scapular, calf, thigh, and abdomen) (Ross and Marfell-Jones 1991) were captured before the first test, while weight was captured before each testing session. Ethical approval for this study was granted by the local Research Ethics Committee, while the principles set out by the Declaration of Helsinki were followed.

### Subjects

In total, 72 cyclists participated in this study; 52 were male cyclists, and 20 were female cyclists. For *Part I*, the reliability study of the LnRMSSD, 16 male cyclists were recruited, while for *Part II,* the relationship between LnRMSSD and training status, 52 male and 20 female cyclists were recruited. The 16 cyclists that were recruited for *Part I* of the study also were recruited for *Part 2* of the study.

### Procedures

The protocol procedures for all participants included a PPO and 40 km TT test, before which the LSCT was performed as a standardized warm-up (Part II). The PPO and 40-km TT tests were performed 72 h apart to ensure sufficient recovery time between the two tests. In addition, a subgroup of cyclists performed the LSCT on six occasions for reliability measurement purposes (Part I).

The LSCT is a submaximal cycle test which is performed based riding at different percentages of heart rate maximum (HR_max_), which is determined during a PPO test (Lamberts 2014; Lamberts et al. 2011b). Within the LSCT, cyclists were asked to cycle at 60% of their personal HR_max_ for 6 min (stage 1), followed by cycling for 6 min at 80% of HR_max_ (stage 2) and cycling for 3 min at 90% of HR_max_ (stage 3). Upon completion of stage 3, cyclists were asked to stop pedalling, sit upright, and not speak for 90 s.

**Fig. 1:**
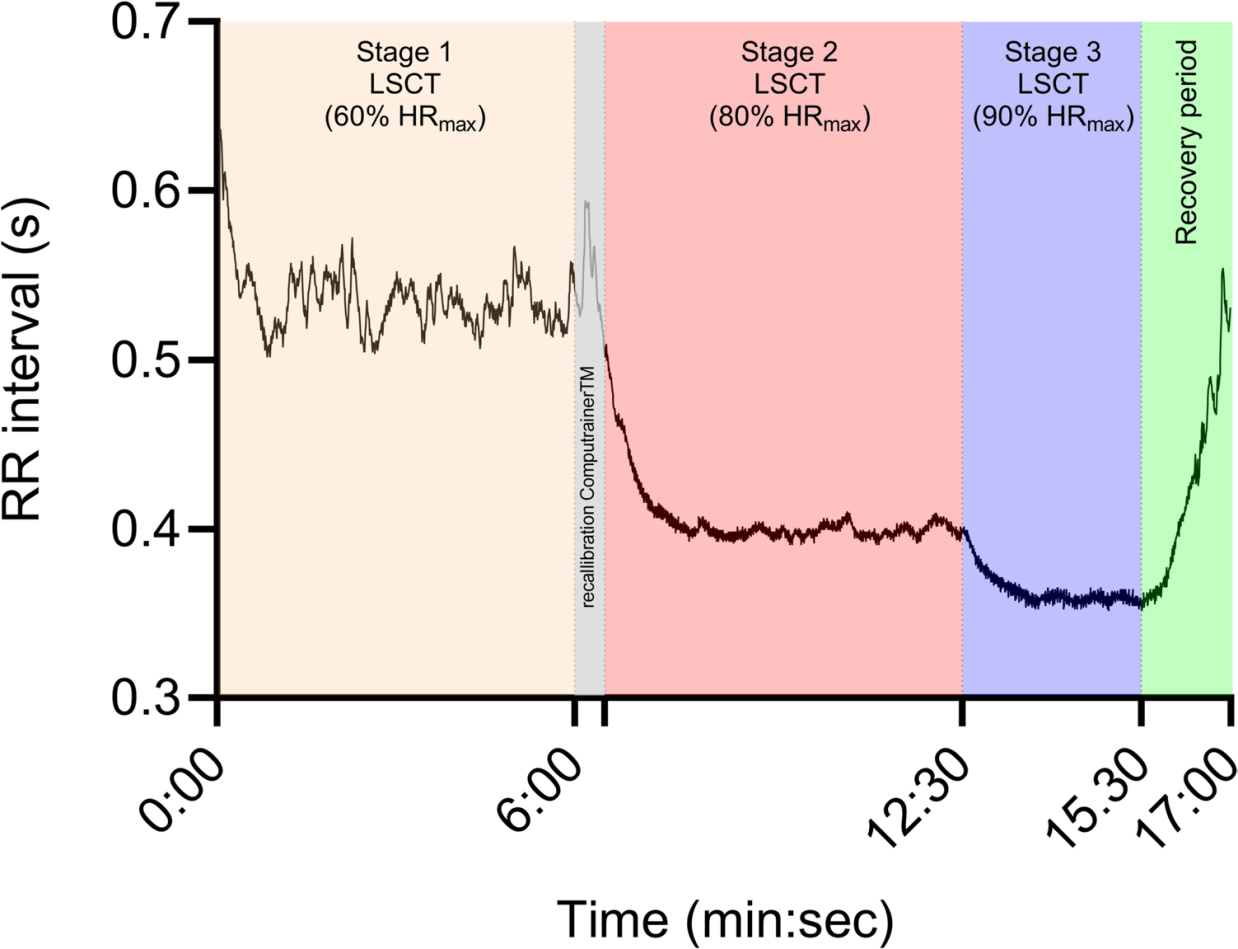
Representation of beat-to-beat (RR) intervals of an arbitrary cyclist during the Lamberts Submaximal Cycle Test (LSCT)

Heart rate and between beat (RR) intervals (see Fig. 1) were measured continuously throughout the LSCT, while recovery LnRMSSD over 30 s (LnRMSSD_30 s_), 60 s (LnRMSSD_60 s_) and 90 s (LnRMSSD_90 s_) were determined during the recovery period. In addition, heart rate recovery over a 60-s period (HRR_60 s_) was also determined and calculated as the difference between the mean heart rate during the last 16 s of stage 3 of and the mean heart rate between 44 and 60 s heart rate during the recovery period, as previously described (Lamberts et al. 2011b; Lamberts 2014; Daanen et al. 2012). A detailed description of the LSCT can in the work of Lamberts et al. (2011b); Lamberts (2014).

The PPO test was started exactly 8 min after completing the LSCT. The starting workload of the PPO for the male cyclist was set at 2.50 W kg^−1^, while the female cyclist started at 2.0 W kg^−1^, after which the workload was increased continuously at a rate of 20 Watts per minute until a cyclist could no longer sustain a cadence greater than 70 revolutions per minute (Lamberts 2014). During the PPO test, respiratory breath-by-breath gas analyses were captured (Oxycon Pro, Viasis, Hoechberg, Germany) and calculated as 15-s averages, while peak oxygen consumption *V*O_2peak_ was determined as the highest 15-s recorded reading. All cyclists were strongly encouraged to perform to the best of their capacity.

The 40 km TT test was started exactly 3 min after completing the LSCT. Cyclists were instructed to complete the 40 km distance as fast as they possibly could. During the 40KM TT, cyclist were not encouraged to get into their own 'zone' and, except for distance completed, were blinded from all other feedback parameters, such as heart rate and power output (Lamberts 2014).

All performance and LCST tests were performed on the cyclist's own bicycle, which was mounted to a CompuTrainer ergometer (CompuTrainer Pro 3D, RacerMate, Seattle, WA, USA). Heart rate and HRV were captured with Suunto heart rate monitors (Suunto T6, Suunto Oy, Vantaa, Finland), which have shown to be able to capture heart rate and HRV data reliably in physically active male and female individuals (Sookan and McKune 2012). In addition, R–R-derived breathing rate was extracted from the Suunto heart rate monitor via the Suunto training software (Montgomery et al. 2009). The load generator of the CompuTrainer was calibrated after the first stage of the LSCT (Fig. 1), as recommended and described before by Lamberts et al. (2011b), Lamberts (2014).

### Reliability of LnRMSSD during recovery (Part I)

For *Part I* of the study, sixteen trained to well-trained male cyclists completed the six LSCTs over a 4-week period. The LSCTs were interspaced by at least 72 h to assure the cyclist could perform the LSCT in a rested state. As mentioned above cyclist were tested at the same time of the day (within 1 h) and under stable and well-controlled environmental conditions (21.7 ± 0.7 °C, 52 ± 4% relative humidity, 101.8 ± 0.7 kPa).

### Relationships between LnRMSSD and training status (Part II)

For *Part II* of the study, 72 cyclists (52 male and 20 female) were completed a both a PPO and 40 km TT test, who were interspaced by a 72-h recovery period. As part of the standardized warm-up before the PPO test and the 40 km TT test, all cyclists performed an LSCT, as also previously described (Lamberts et al. 2011b; Lamberts 2014). PPO, *V*O_2_peak and 40 km TT time were used indicators of the training status of the cyclists (Jeukendrup et al. 2000).

### Data processing and analyses

Heart rate and HRV were captured continuously using a Suunto T6 heart rate monitor (Suunto Oy, Vantaa, Finland). Heart rate data were downloaded and analyzed with Suunto Training Manager (Version 2.1.0.3, Suunto Oy, Vantaa, Finland). Heart rate files were exported to a Suunto STE file (Suunto Training Manager) and imported into Kubios HRV software (Kubios HRV, Version 2.2, Kuopio University, Kuopio, Finland) for analyses. Occasional ectopic beats (irregularity of heart rhythm involving extra or skipped heart beats; namely, extra-systolic and consecutive compensatory beats) were visually identified and, if necessary, corrected by a Kubios HRV filter. RMSSD data were logged transformed (LnRMSSD) and calculated over the first 30 s (LnRMSSD_30 s_; 15.30–16.00 (min.s)), 60 s (LnRMSSD_60 s_; 15.30–16.30 (min.s) and 90 s (LnRMSSD_90 s_; 15.30–17.00 (min.s)) after completing stage 3 of the LSCT. To correct for differences in elicited heart rate during the recovery period (van den Berg et al. 2018; de Geus et al. 2019), corrected LnRMSSD (cLRMMSD) were calculated based on mean heart rate during the 30 s, 60 s and 90 s recovery periods. Corrected RMSSD (cRMSSD) was calculated as cRMSSD = RMSDD e^−0.3243 (60 –heart rate)^, as recommended by van den Berg et al. (2018). Once cRMSSD was determined, the data were logged transformed to calculate cLnRMSSD. HRR_60 s_ was calculated as the mean difference between the last 16 s of stage 3 of the LSCT and the mean heart rate between 44 and 60 s into the recovery period, as previously described (Lamberts et al. 2011b; Daanen et al. 2012).

### Statistical analyses

Statistical analysis was performed using STATISTICA version 13.5.0.17 (Stat-soft Inc., Tulsa, OK, USA). The normality of the data was checked with a Kolmogorov–Smirnov test. All data are expressed as mean ± standard deviation (mean ± SD). The reliability of the LnRMSSD and HRR measurements after stage 3 of the LSCT *(Part I)* were analyzed by intraclass correlation coefficient (ICC) and the 95% confidence intervals. In addition, the typical error of the measurements (TEM) and the TEM percentage (TEM%) were also calculated using a spreadsheet downloaded from www.sportsci.org, and expressed as mean and 90% confidence intervals. Relationships between cycling performance (*V*O_2peak,_ PPO and 40-km TT time) and LnRMSSD and HRR *(Part II)* were studied with Pearson product-moment correlations, while the standard error of the estimate was also determined (GraphPad Prism version 9.5.1). Differences in relationships between male and female cyclists were studied by analyzing the slope and *y*-axis intercepts of the relationships. Significance was set to *p* < 0.05.

## Results

The data of 2 cyclists, who participated in the reliability study (*Part* *I*) and relationship study (*Part* *II*), were excluded for further analyses. One cyclist could not complete all trials, while another cyclist used medication that could have influenced cycling performance (Lamberts et al. 2011b). In total, 50 male cyclists, with a training history of 8 ± 7 years and on average trained 10 ± 4 h per week, and 20 female cyclists, with a training history of 7 ± 4 years and on average trained 9 ± 3 h per week, completed the study. The descriptive and performance characteristics of these 50 male and 20 female cyclists are shown in Table 1. Based on the range in PPO (males: 275–474; females: 254–364W) and *V*O_2peak_ values (males: 46.5–76.8 ml min kg^−1^; females 43.9–56.4 ml min kg^−1^) the cyclist in our study categorized from 'trained' to 'elite' cyclists (De Pauw et al. 2013; Decroix et al. 2016).

**Table 1:** Descriptive and performance data of the 14 cyclists who participated in the reliability study, while 70 cyclists patriated in the relationship study

Variable	Reliability study	Relationship study	
	Male cyclists (*n* = 14)	Female cyclists (*n* = 20)	Male cyclists (*n* = 50)
Descriptive data			
Age (years)	31 ± 4	30 ± 7	31 ± 7
Height (cm)	182 ± 8	168 ± 6	181 ± 8
Body mass (kg)	77.0 ± 5.8	60.8 ± 6.6	76.1 ± 7.2
Body fat percentage (%)	15.7 ± 2.7	20.1 ± 3.3	13.9 ± 2.7
Sum of skinfolds (mm)	63.9 ± 12.0	68.0 ± 15.9	64.3 ± 13.5
Performance data			
*V*O_2max_ (ml min^−1^ kg^−1^)	57.5 ± 6.0	50.5 ± 3.4	58.5 ± 6.3
*V*O_2max_ (L min^−1^)	4.4 ± 0.6	3.1 ± 0.3	4.4 ± 0.5
PPO (W)	379 ± 46	288 ± 25	383 ± 33
PPO (W kg^−1^)	4.9 ± 0.4	4.8 ± 0.3	5.1 ± 0.6
40 km TT time (min.s)	66.08 ± 3.17	73.46 ± 2.30	65.31 ± 2.46
40 km TT power (W)	253 ± 35	181 ± 20	259 ± 30

The data are expressed as mean ± standard deviation

*VO*_*2max*_* maximum oxygen consumption, PPO peak power output; 40 km TT 40 km time trial*

### Reliability of LnRMSSD during recovery (Part I)

Heart rate at the end of the LSCT, from which the LnRMMDS measurements were performed, as well as the mean breathing rate during the recovery period, are shown in Table 2. The reliability of LnRMSSD and the corrected LnRMSSD for mean heart rate over 30-, 60- and 90-s recovery periods are shown in Table 3. The highest reliability was found in LnRMSSD_60 s_ (ICC = 0.97, TEM% = 5.8%), which was similar to HRR_60 s_ (ICC = 0.97, TEM% = 4.3%). Substantially poorer reliability values were found for LnRMSSD_30 s_ (ICC = 0.84, TEM% = 17.2%) and LnRMSSD_90 s_ LnRMSSD_90 s_ (ICC = 0.94, TEM% = 9.5%). In addition, LnRMSSD corrected for heart rate (caLnRMSSD did not improve the reliability of the LnRMSSD measurement (see also Table 3).

**Table 2:** Reliability of submaximal heart rate and breathing rate during stage 3 and the recovery period of the LSCT

	LSCT_1_	LSCT_2_	LSCT_3_	LSCT_4_	LSCT_5_	LSCT_6_	Mean	ICC	TEM	TEM (%)
Heart rate from whichLnRMSSD and BR was measured										
Mean HR stage 3 _2 min_ (bpm)	172 ± 4	171 ± 4	171 ± 4	172 ± 4	172 ± 5	171 ± 4	171 ± 4	0.96 (0.92–0.98)	0.9 (0.8–1.0)	0.5 (0.4–0.6)
Percentage of HR_max_ (%)	90 ± 1	90 ± 1	90 ± 1	90 ± 1	90 ± 1	90 ± 1	90 ± 1	**–**	**–**	**–**
Mean HR stage 3 _1 min_ (bpm)	172 ± 4	171 ± 3	171 ± 4	171 ± 4	172 ± 5	171 ± 4	171 ± 4	0.94 (0.89–0.98)	1.0 (0.9–1.2)	0.6 (0.5–0.7)
Percentage of HR_max_ (%)	90 ± 1	90 ± 1	90 ± 0	90 ± 1	90 ± 0	90 ± 1	90 ± 0	**–**	**–**	**–**
Breathing rate										
Mean BR_stage 3—60 s_ (brpm)	47 ± 5	46 ± 5	46 ± 4	47 ± 5	47 ± 5	48 ± 5	47 ± 4	0.82 (0.70–0.92)	2.2 (1.9–2.5)	4.7 (4.1–5.6)
Mean BR_recovery—30 s_ (brpm)	40 ± 4	40 ± 5	41 ± 5	40 ± 4	40 ± 3	40 ± 4	40 ± 4	0.65 (0.46–0.83)	2.6 (2.3–3.1)	6.8 (5.9–8.0)
Mean BR _recovery—60 s_ (brpm)	36 ± 3	36 ± 3	35 ± 2	36 ± 2	36 ± 2	36 ± 3	36 ± 2	0.65 (0.46–0.83)	1.6 (1.4–1.9)	4.5 (3.9–5.3)
Mean BR _recovery—90 s_ (brpm)	32 ± 4	32 ± 5	31 ± 3	31 ± 3	31 ± 3	32 ± 3	31 ± 3	0.50 (0.29–0.73)	2.7 (2.3–3.2)	9.0 (7.8–10.7)

*LSCT *Lamberts Submaximal Cycle Test,* ICC *intraclass correlation,* TEM *Typical Error of the Measurement,* TEM (%) *Typical Error of the Measurement percentage as a CV*, HR *heart rate HR_max_ maximum heart rate*, HR stage 3*_*2min*_ heart rate during the last 2 min of stage 3 of the LSCT*, HR stage 3 *_*1min*_ heart rate during the last minute of stage 3 of the LSCT*, bpm *beats per minute*, BR*_*stage 3*_ breathing rate during the last 60 s of stage 3 of the LSCT*, BR*_recovery—30 s_ breathing rate during the first 30 of the recovery period, *BR*_recovery—60 s_ breathing rate during the first 60 of the recovery period, *BR*_recovery—90 s_ breathing rate during the first 90 of the recovery period, *brpm breaths per minute*

**Table 3:** Mean physiological changes and the reliability of the heart from rate at the end of stage 3, breathing rate, LnRMSSD_30 s_, LnRMSSD_60_ and LnRMSSD_90 s_ and HRR within the 6 LSCTs

	LSCT_1_	LSCT_2_	LSCT_3_	LSCT_4_	LSCT_5_	LSCT_6_	Mean	ICC	TEM	TEM (%)
LnRMSSD										
LnRMSSD_30 s_ (ms)	1.10 ± 0.32	1.06 ± 0.37	1.05 ± 0.37	1.04 ± 0.33	1.04 ± 0.30	0.99 ± 0.37	1.05 ± 0.34	0.84 (0.62–0.93)	0.13 (0.12–0.16)	17.2 (14.9–20.4)
LnRMSSD_60 s_ (ms)	1.16 ± 0.35	1.19 ± 0.32	1.18 ± 0.35	1.18 ± 0.36	1.18 ± 0.36	1.19 ± 0.37	1.18 ± 0.35	0.97 (0.92–0.99)	0.06 (0.05–0.07)	5.8 (5.1–6.8)
LnRMSSD_90 s_ (ms)	1.25 ± 0.45	1.33 ± 0.51	1.29 ± 0.48	1.29 ± 0.41	1.28 ± 0.40	1.29 ± 0.45	1.29 ± 0.45	0.94 (0.84–0.98)	0.13 (0.11–0.15)	9.5 (8.2–11.1)
Corrected LnRMSSD for heart rate										
cLnRMSSD_30 s_ (ms)	3.55 ± 0.42	3.41 ± 0.42	3.62 ± 0.37	3.49 ± 0.49	3.57 ± 0.65	3.48 ± 0.47	3.50 ± 0.38	0.92 (0.85–0.97)	0.31 (0.27–0.37)	10.4 (9.0–12.3)
cLnRMSSD_60 s_ (ms)	3.65 ± 1.05	3.68 ± 0.93	3.59 ± 0.99	3.64 ± 1.05	3.66 ± 1.00	3.71 ± 0.93	3.66 ± 0.97	0.97 (0.93–0.98)	0.21 (0.18–0.25)	7.4 (6.4–8.8)
cLnRMSSD_90 s_ (ms)	3.68 ± 1.11	3.66 ± 1.04	3.72 ± 1.13	4.06 ± 1.08	3.62 ± 0.91	3.79 ± 1.01	3.76 ± 0.95	0.82 (0.69–0.92)	0.48 (0.42–0.57)	13.6 (11.8–16.2)
Heart rate recovery										
HRR_60 s_ (beats)	45 ± 10	44 ± 10	45 ± 9	44 ± 10	45 ± 9	45 ± 9	44 ± 9	0.97 (0.91–0.99)	1.7 (1.5–2.0)	4.3 (3.8–5.1)

*LSCT *Lamberts Submaximal Cycle Test*, ICC *intraclass correlation*, TEM *Typical Error of the Measurement*, TEM (%), *Typical Error of the Measurement percentage as a CV*, ms *milliseconds*,* *LnRMSSD*_*30 s*_ LnRMSSD during the first 30 s of the recovery period, *LnRMSSD*_*90 s*_ LnRMSSD during the first 90 s of the recovery period, *LnRMSSD*_*30 s*_ LnRMSSD during the first 30 s of the recovery period, *cLnRMSSD* corrected LnRMSSD for heart rate

### Relationships between LnRMSSD and training status (Part II)

Relationships between LnRMSSD and the training status of the cyclists were strongest between LnRMSSD_60 s_ and *V*O_2peak_ (males: *r* = 0.71; females: *r* = 0.63), PPO (males: *r* = 0.93; females: *r* = 0.85) and 40 km TT (males: *r* = – 0.87; females: *r* = – 0.63) (Fig. 1). Slightly weaker relationships were found between LnRMSSD_90 s_ and *V*O_2peak_ (males: r = 0.62; females: *r* = 0.67), PPO (males: *r* = 0.79; females: *r* = 0.68) and 40 km TT (males: *r* = – 0.78; females: *r* = – 0.64). Even weaker relationships were found between LnRMSSD_30 s_ and *V*O_2peak_ (*r* = 0.54), PPO (r = 0.75) and 40 km TT (*r* = – 0.60) in male cyclists, while relationships were found between LnRMSSD_30 s_ and training status in female cyclists. Differences in the LnRMSSD_60 s_ and training status in male and female cyclists in all relationship analyses were characterized by different *Y*-axis intercepts (*p *< 0.001), while the slope angle was similar in male and female cyclists.

## Discussion

To our knowledge, this is the first study to determine the reliability of LnRMSSD after a standardized submaximal test and its relationship with training status in trained to elite cyclists.

### Reliability of LnRMSSD during recovery (Part I)

Although the variation in LnRMSSD during the recovery period was very small due to high exercise intensity (90% HR_max_), LnRMSSD could be measured reliably after the LSCT. The highest reliability was found for the LnRMSSD_60 s_, with an ICC of 0.97 and a TEM% of 5.8%, which was substantially better than the LnRMSSD measurements over 30 and 90 s. The TEM% of 5.8% of LnRMSSD_60 s_ in this study is in line with the findings of Al Haddad et al. (2011), who reported a TEM% of LnRMMSD_5min_ of 5.9% after a submaximal running protocol.

Similar heart and breathing rates at the end of the test and during the recovery have likely contributed substantially to the good reliability values we found. This needs to be taken into account as we hypothesize that the reliability of LnRMSSD might be substantially lower in protocols that fix other physiological parameters, such as power, and in which submaximal heart and breathing rates vary more. Although LnRMSSD, in our case, did not further improve the reliability of the LnRMSSD (likely due to very similar submaximal heart rates (Table 2), correcting for submaximal heart rate in protocols in which heart rate varies more might be helpful (van den Berg et al. 2018; de Geus et al. 2019).

The successful measurement of LnRMSSD over ultra-short time frames as in our study is supported by Munoz et al. (2015), who reported strong correlations between the traditional 4 and 5 min LnRMSSD measurements and ultra-short 30 s LnRMSSD (*r* = 0.932) and 120 s LnRMSSD (*r* = 0.986). In contrast to Munoz et al. (2015), who reported more robust relationships with 120 s than 30 s LnRMSSD measurements, we observed slightly poorer reliability and relationships with training status and longer LnRMSSD measurements. This can likely be attributed to the higher variation in heart and breathing rates that were observed during the KnRMSSD90 s measurement (Table 3).

### Relationships between LnRMSSD and training status (Part II)

**Fig. 2:**
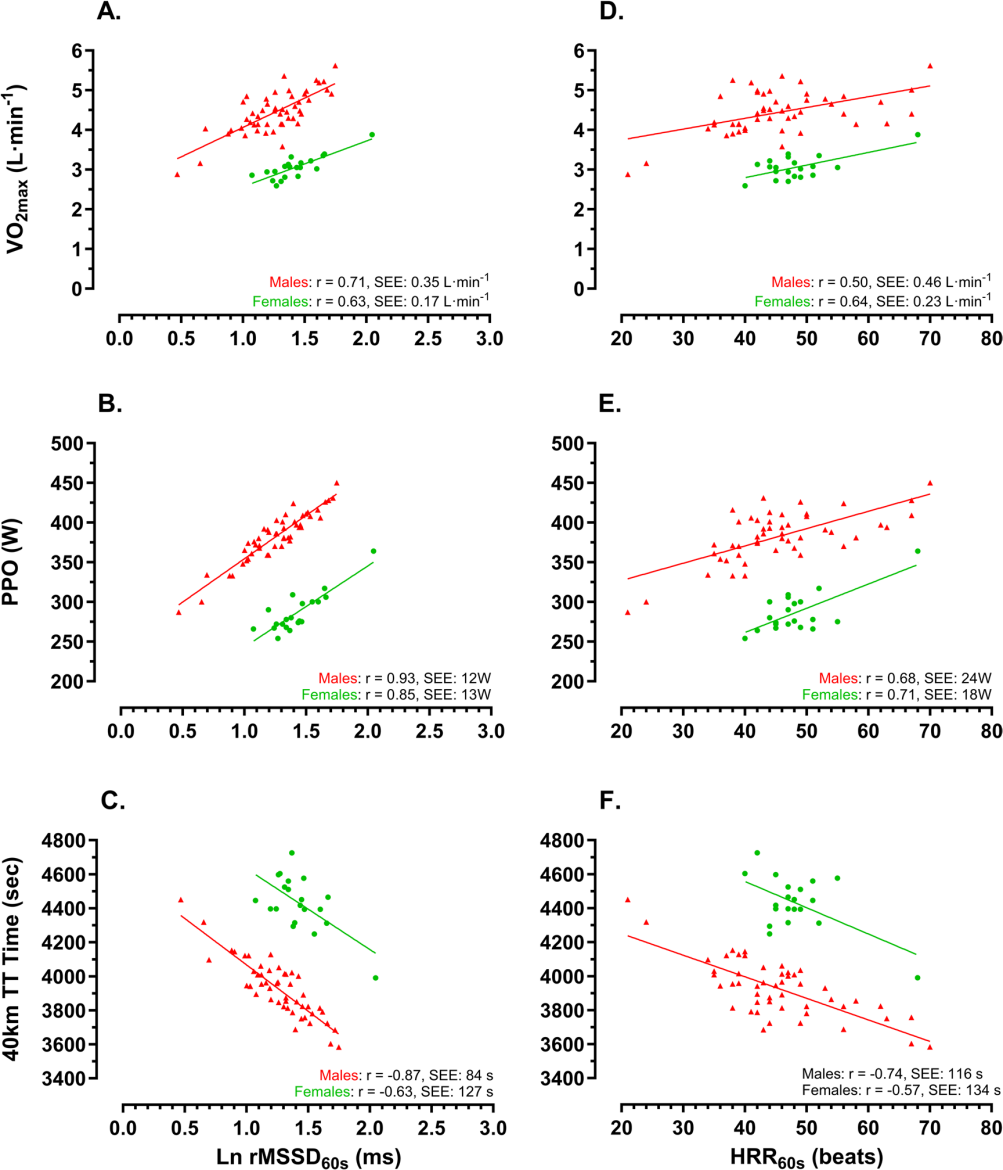
Relationships between training status based on maximal oxygen consumption (*V*O_2peak_), Peak Power Output (PPO) and 40 km time trail time (40 km TT) and LnRMSSD_60 s_ (**A**–**C**) and HRR_60 s_ (**D**–**F**)

The strongest relationship with the training status of the cyclist was found with LnRMSSD_60 s_, and which were slightly better than with HRR_60 s_ (Fig. 2). The weaker [LnRMDDS_30 s_ and LnRMSSD_90 s_ (males)] and in females, no relationships that were found between training status LnRMSSD_90 s_, can likely be explained by the larger variability in the LnRMSSD measurements, as can be seen in *Part I* of this study (see also Fig. 2).

Although similar slopes were found for male and female cyclists between LnRMSSD_60 s_ and HRR_60 s_ and cycling performance parameters, *Y*-axis intercepts differed significantly between male and female cyclists. This finding indicates a similar relationship exists between training status and LnRMSSD. However, training status indicators, such as PPO, *V*O_2peak_ and 40-km TT time, are lower in female than male cyclists. This is supported by the work of Valenzuela et al. (2022) and Mateo-March et al. (2022), who have reported lower power output profiles in female than in male professional cyclists. Differences in power output can partially be explained by the difference in body composition between male and female cyclists, with female cyclists having lower muscle mass and higher body fat percentages than their male counterparts (Lamberts and Davidowitz 2014).

The slightly weaker relationships found in the female cyclist can, on the one side, be explained by the smaller sample size, while on the other side, female cyclists might be less accustomed to these performance tests and, therefore, might have slightly more variability in their outcome measures than male cyclists. However, with female cycling becoming rapidly more professional (Mateo-March et al. 2022; van Erp and Lamberts 2022; Lamberts and Davidowitz 2014; Sanders et al. 2019; van Erp et al. 2019), we expect this difference to rapidly become smaller and similar to male cyclists in the near future.

Overall, slightly stronger relationships were found between training load and LnRMSSD_60 s_ than with HRR_60 s_. This can possibly be explained by the fact that HRR_60_s tend to respond very quickly to changes in training load, resulting in slightly more variation, while traditionally, heart rate variability is believed to reflect the long-term modulation of the autonomic nervous system (Buchheit et al. 2007, 2008). However, this raises the question of whether LnRMSSD_60 s_ can reflect sudden changes in training status and what role it can play in monitoring and optimizing training prescription. In support of LnRMSSD, Javaloyes et al. (2020, 2018) recently showed that prescribing training based on LnRMSSD is likely to result in better performance enhancements than other traditional training methods.

Although the findings of this study are promising and suggest that recovery LnRMSSD_60 s_ can play an important role in monitoring cyclists, the current study also has some limitations. The first thing we need to acknowledge is that submaximal heart rates and breathing rates were very similar within our population group (Table 2), which likely has positively contributed to our LnRMSSD reliability findings. The similar heart and breathing rates can be explained by the unique design of the LSCT, which requires cyclists to ride at 60, 80 and 90% of their personal maximal heart rate. In submaximal protocols requiring athletes to exercise based on other physiological parameters, such as power or running speed, heart and breathing rate might vary more, potentially resulting in substantially poorer LnRMSSD reliability values, In protocols that elicited varying submaximal heart rates, correcting for mean heart rate might be worthwhile as suggested by van den Berg et al. (van den Berg et al. 2018) and de Geus et al. (2019).

Another limitation of this study is that breathing rate was estimated from the Suunto heart rate monitors rather than measured directly. To our knowledge, no studies have validated this predicted breathing rate from Suunto heart rate monitors; therefore, these data need to be interpreted cautiously. Future research needs to confirm our findings and establish whether smartwatches' can reliably measure breathing rates.

Last but not least, the current study shows that there is that good relationships exist LnRMSSD_60 s_ and performance parameters, such as PPO, *V*O_2peak_ and 40 km TT time. Although these findings are promising from a monitoring point of view, it is still unknown if a change in training status is directly reflected by a change in LnRMSSD_60 s_. Traditionally, changes in HRV reflect neuromodulation of the autonomic nervous system and, therefore, is relatively slow. However, LnRMSSD60 s measured directly after exercise mainly reflects parasympathetic reactivation, similar to HRR. Therefore, LnRMSSD_60 s_ might be able to reflect changes in training status quite quickly, such as HRR, but future research needs to confirm this hypothesis.

In conclusion, the findings of this study show that LnRMSSD_60 s_ can be measured reliably during the recovery period of the LSCT. Important to acknowledge here is that cyclists elicited similar absolute and relative submaximal heart rates that were associated with similar breathing rates. This likely contributed positively to the reliability of LnRMSSD_60 s_ and suggests LnRMSSD_60 s_ might be less reliable when measured after protocols clamping other variables, such as power. In line with the reliability, the strongest relationships between LnRMSSD and training status were found with LnRMSSD_60 s_. Although these findings are promising and suggest that LnRMSSD might become an important monitoring parameter, future research needs to confirm the capacity of LnRMSSD to reflect a change in training status.

### Practical application

This paper shows that LnRMSSD_60 s_ can be measured reliably after the LSCT, which partially can be explained by the unique design that requires cyclists to ride at 60, 80 and 905 of their heart rate maximum. In addition, good relationships were found between LnRMSSD_60 s_ and PPO, *V*O_2peak_ and 40 km TT time, suggesting that these parameters can be predicted from LnRMSSD_60 s_ in trained-to-elite cyclists. Last but not least, LnRMSSD_60 s_ shows potential to become an important parameter in monitoring and fine-tuning training prescriptions in trained-to-elite cyclists. However, future research needs to confirm how well LnRMSSD_60 s_ can reflect changes in training status.

## Data Availability

The datasets generated during the current study are available from the corresponding author on reasonable request.
